# A novel apidaecin Api-PR19 synergizes with the gut microbial community to maintain intestinal health and promote growth performance of broilers

**DOI:** 10.1186/s40104-020-00462-1

**Published:** 2020-06-17

**Authors:** Shengru Wu, Jian Wang, Liqin Zhu, Hao Ren, Xiaojun Yang

**Affiliations:** College of Animal Science and Technology, Northwest A&F University, Yangling, Shaanxi China

**Keywords:** 16S rRNA gene sequencing, Antibiotic growth promoters, Apidaecin, Broiler chickens, Growth performance, Gut microbiota, Immune

## Abstract

**Background:**

Antibiotic growth promoters (AGPs) have been used as growth promoters to maintain animal intestinal health and improve feed efficiency in broilers by inhibiting pathogen proliferation. In view of the growing emergence of antibiotic-resistant pathogen strains and drug residue issues, novel treatments are increasingly required. This study aimed to compare two antimicrobial approaches for managing pathogen infection and maintaining animal intestinal health in broilers by supplying Apidaecin Api-PR19 and AGPs over 42 d of a feeding trial.

**Results:**

Compared with the broilers that were only fed a corn-soybean basal diet (CON group), supplementation with Api-PR19 and AGP (respectively named the ABP and AGP groups) both increased the feed conversion efficiency. When compared with the AGP group, Api-PR19 supplementation could significantly increase the organ index of the bursa of fabricius and subtype H9 antibody level in broiler chickens. Moreover, when compared with the CON group, the intestinal villus height, intestinal nutrient transport, and intestinal sIgA content were all increased in the Api-PR19 group, while AGP supplementation was harmful to the intestinal villus height and intestinal nutrient transport. By assessing the antibacterial effect of Api-PR19 and antibiotics *in vitro* and *in vivo*, we found that Api-PR19 and antibiotics both inhibited the growth of pathogens, including *Escherichia coli* and *Campylobacter jejuni*. Furthermore, by using 16S rRNA gene sequencing, the beneficial bacteria and microbiota in broilers were not disturbed but improved by apidaecin Api-PR19, including the genera of *Eubacterium* and *Christensenella* and the species of *uncultured*_*Eubacterium*_*sp*, *Clostridium*_*asparagiforme*, and *uncultured*_*Christensenella*_*sp*, which were positively related to improved intestinal development, absorption, and immune function.

**Conclusion:**

Apidaecin Api-PR19 treatment could combat pathogen infection and had little negative impact on beneficial bacteria in the gut compared to antibiotic treatment, subsequently improving intestinal development, absorption, and immune function.

## Introduction

The gastrointestinal tract is colonized by a diverse microbiota, which has increasingly been associated with “intestinal” or “non-intestinal” diseases [1–3]. Conditioned pathogen infections, such as those caused by enteropathogenic *Escherichia coli* (*E. coli*), enteroinvasive *E. coli*, enterohemorrhagic *E coli*, intestinal adhesive *E. coli*, and Shiga toxin-producing *E. coli*, *Campylobacter*, *Salmonella,* and *Clostridium perfringens*, are one of the main causes of intestinal inflammation diseases in broilers [4, 5]. Gastrointestinal pathogen infections in broilers serve as an important cause of human gastrointestinal pathogen infections and can be harmful to human and broiler health [6, 7]. Alterations of the microbiota and excessive proliferation of pathogenic bacterium may contribute to many chronic and degenerative diseases in broilers and humans, including necrotic enteritis and inflammatory bowel diseases [8, 9]. A balanced and healthy gut microbiota not only utilizes the functional metabolic cycle to provide some necessary nutrients to the host but also trains the immune system to detect pathogens and prevent dysbiosis of the gut microbiota [10, 11]. Thus, it is critical to identify how a balanced gut microbiota functions and how it is influenced by various factors, especially when developing approaches for maintaining intestinal homeostatic balance and health.

Antibiotic growth promoters (AGPs) have been used as growth promoters for a long time by maintaining gut health and improving feed efficiency [12, 13]. The use of AGPs in animal feeding has been gradually forbidden due to their negative effects, such as destruction of the healthy intestinal microbiota, antimicrobial resistance, and drug residue issues [14, 15]. However, the forbidden use of AGPs may increase conditioned pathogen infection risk in broilers, which could further influence the infection risk in humans [16]. The increasing emergence of conditioned pathogens that are resistant to many currently available antibiotics, and the negative effects of AGPs on the healthy intestinal microbiota, structure, and function have recently attracted more attention. These conditions highlight the need to identify novel alternatives and complements to AGPs for effective and green special additives, which can both stimulate the productive potential and maintain the intestinal health of broilers [17]. Antibacterial peptides (ABPs), produced by bacteria, insects, amphibians, fishes, mammals, and even plants upon pathogen infection, as well as by chemical synthesis or *in vitro* microbial fermentation using gene engineering strains, are possible candidates for the design of new antimicrobial agents because of their natural antimicrobial properties and a low propensity for development of resistance by microorganisms [18].

Based on their secondary structure with a positive charge and amphipathic properties, ABPs can exert their antibacterial roles by affecting the cytomembrane of bacterium, or by influencing bacterial transcription and translation processes and therefore inducing metabolic death of the bacterium [19]. Of these, apidaecins HbIa, HbIb and HbII are a series of small, proline-rich (Pro-rich), 18- to 20-residue peptides produced by the hemolymph of insects [20]. Honeybee-derived apidaecins are lethal to many Gram-negative bacteria, such as *E. coli*, *Enterobacter cloacae*, *Klebsiella pneumonia*, *Salmonella typhimurium*, *Salmonella typhi*, *Shigella dysenteriae*, *Acinetobacter calcoaceticus* and *Agrobacterium tumefaciens*, through a bacteriostatic rather than a lytic process [21]. In brief, the mechanism of action by which apidaecins kill bacteria involves an initial, non-specific encounter of the peptide with an outer membrane component, followed by invasion into the periplasmic space. Apidaecins then cross the inner membrane by a specific and essentially irreversible engagement with a receptor/docking molecule. In the final step, the peptide is translocated into the interior of the cell where it meets its ultimate target and then performs it bacteriostatic function. For instance, it can inhibit two main functions of DnaK (an *E. coli* HSP70): ATPase activity and protein folding [22]. Compared with antibiotics, the immediate effect, apparent nontoxicity toward eukaryotic cells, and little or no bacterial resistance of apidaecins have been suggested [22]; therefore, recombinant apidaecins have been widely successfully expressed and produced in *Streptomyces* sp., *Lactococcus lactis*, and *Pichia pastoris* expression system [23]. Hence, apidaecins could serve as one such potential alternative to antibiotics in the swine and poultry industries.

Several ABPs, such as cecropin, β defensins, AMP-P5, AMP-A3, and apidaecins, have also attracted increased attention from the poultry industry due to their beneficial effects on growth performance and health in animals as well as their abilities to reduce the conditioned pathogen infection risk in humans who eat these animal products [18]. However, the effect of apidaecins on the gut microbiota of broilers remains unclear, which could help to better understand their roles in pathogen infection defense, maintenance of gut health, and promotion of broiler growth. In this study, we used the recombinant apidaecin Api-PR19 (designed based on the first identified apidaecin HbIb and another recombinant apidaecin Hb1C-20) as a substitution for AGPs. The Api-PR19 was produced by engineered prokaryotic expression bacteria in which only a proline was added to the N-terminus of peptide 1C-20, demonstrating the strongest anti-bacterial ability, according to a previous study [20]. Moreover, we investigated how the gut microbiota changes in the presence or absence of antibiotic and apidaecin.

## Materials and methods

### Apidaecin Api-PR19 and antibiotics

The apidaecin Api-PR19 was kindly provided by Aolinberer (Gansu, China) and is the subject of Chinese patents ZL2014–1-0654343.X. The details regarding Apidaecin Api-PR19 are listed in Table S1. In brief, Api-PR19 is an arginine- and proline-rich peptide, forming a stable polyproline helical structure and exposing the guanidine group of arginine to contact the surface of gram-negative bacteria. Enramycin was used as the positive antibiotic control in the present study. The bacteria used in the minimum inhibitory concentration (MIC) assay included *Escherichia coli* ATCC25922, *Salmonella typhimurium* ATCC14028, *Staphylococcus aureus* ATCC25923, *Helicobacter pylori* ATCC43504, and *Pasteurella* ATCC19427, which were purchased from the American Type Culture Collection (ATCC; Rockville, MD, USA).

### Antimicrobial activity tests for apidaecin Api-PR19 *in vitro*

By using the minimal inhibitory concentration (MIC) assay depending on a microtiter broth dilution method [24], the MIC of Api-PR19 for inhibiting bacterial growth included *Escherichia coli* ATCC25922, *Salmonella typhimurium* ATCC14028, *Staphylococcus aureus* ATCC25923, *Helicobacter pylori* ATCC43504, and *Pasteurella* ATCC19427. The MIC was defined as the lowest concentration of apidaecin Api-PR19 required to inhibit growth of the test bacterium. In brief, agar dilution involved the incorporation of different concentrations of the antimicrobial substance into a nutrient agar medium followed by the application of a standardized number of cells to the surface of the agar plate. For broth dilution, often determined in a 96-well microtiter plate format, bacteria were inoculated into a liquid growth medium in the presence of different concentrations of an antimicrobial agent. Growth was assessed after incubation for a defined period of time (16–20 h), and the MIC value was read. Three replications (*n* = 3) of each treatment were performed.

Furthermore, Oxford cup methods were used to exam and compare the antibacterial activity of ciprofloxacin and Api-PR19. We added 0.1 mL of diluted inoculum (10^5^ CFU/mL) from *Escherichia coli* ATCC25922 to the surface of warm nutrient agar (NA)/SD agar plates (90 × 15 mm) with the help of a sterile cotton swab, and then allowed it to solidify. Sterilized Oxford cups (Ф 5 mm) were then placed on the agar medium and filled with 200 μL of Api-PR19 solution (200 mg/mL), and 200 μL of ciprofloxacin solution (200 mg/mL) was used as a control. The plates were incubated for 18 h at 37 °C. The anti-microbial activity was evaluated by comparing the diameter zone of transparent inhibition against *E. coli* ATCC25922.

Moreover, by further using *Escherichia coli* ATCC25922 as the indicator, the time effect (different time points included 1, 2, 4, 8, and 16 h after treatment) of different concentrations (0.25, 1, and 10 times of the MIC) of Api-PR19 on the inhibition of *E. coli* ATCC25922 proliferation was further tested in accordance with a previous study [25]. Three replications (*n* = 3) of each treatment were performed.

### Stress resistance tests of apidaecin Api-PR19 *in vitro*

The effect of different factors, including enzymes (pepsin, trypsin, and α-chymotrypsin), pH and temperature, on Apidaecin Api-PR19 stability were evaluated in accordance with previously described methods [25]. First, aliquots of 20 μg/L apidaecin Api-PR19 in PBS (pH 7.4) were separately treated with protease [pepsin (3,000 NFU, Sigma), trypsin (250 NFU, Sigma), and α-chymotrypsin (1,200 U, Sigma)] at a substrate: protease molar ratio of 300:1 at 37 °C for 30 min, and then 100 μL of the treated Apidaecin Api-PR19 solution was further used for the antimicrobial activity tests *in vitro.* Aliquots of 20 μg/L apidaecin Api-PR19 in PBS (pH 7.4) were then separately treated under gradient pH values (pH 2.0, 3.0, 4.0, 5.0, 6.0, 7.0, 8.0, and 9.0, the pH was adjusted using 1 mol/L HCl or NaOH solution, and the concentration of Api-PR19 were also adjusted to 20 μg/L by adding some extra Api-PR19 when considering the increased volume of HCL or NaOH solution) at 37 °C for 30 min. Next, 100 μL of the treated apidaecin Api-PR19 solution was further used for antimicrobial activity tests *in vitro*. Third, aliquots of 20 μg/L apidaecin Api-PR19 in PBS (pH 7.4) were separately treated under gradient temperatures (20, 30, 40, 50, 60, 70, 80 and 90 °C) for 30 min, and then 100 μL of the treated apidaecin Api-PR19 solution was further used for antimicrobial activity tests *in vitro*. After the treatment, the indicator bacterial solution (*Escherichia coli* ATCC25922, 5 × 10^5^ CFU/mL) was mixed with the collected 100 μL treated peptide solution and then incubated at 37 °C for 16 h. Simultaneously, a similar reaction system at 37 °C (pH 7.0) was established as positive control; the same systems without apidaecin Api-PR19 were correspondingly used as a negative control. Three replications (*n* = 3) of each treatment were performed. After incubation, the OD value of the positive control (A2), negative control (A1), and test groups (A) were recorded, and then the bactericidal efficiency of Api-PR19 after the stress challenge (enzymes, pH and temperatures) tests was calculated using (A−A1) / (A1−A2) × 100%.

### Birds and experimental design

All the birds and experimental protocols in this study were approved by the Institution Animal Care and Use Committee of Northwest A&F University. Based on a single factor experimental design, a total of 630 one-day-old Arbor Acres male broilers were randomly assigned to 5 groups with 7 replications and 18 birds per replicate, including 5 treatments: the control (CON) group was supplied with a corn-soybean basal diet, the antibiotics (AGP) group was supplied with an additional 4.96 mg/kg enramycin, and the other 3 apidaecin Api-PR19 (AP1, AP2, and AP3) groups were supplied with an additional 100, 200, and 300 mg/kg apidaecin Api-PR19. The basal diet was typical of diets commonly used in the Northwestern District of China to meet National Research Council (NRC, USA, 1994) recommendations (Table S2). All chickens were kept in an environmentally controlled henhouse with double-floor metabolism cages and exposed to a 24-h photoperiod. Water and feeds were available ad libitum. The brooding temperature was maintained close to their requirements. Moreover, the details of the broilers immunization program were in accordance with the procedures recommended by the Ministry of Agriculture of China and are listed in Table S3. The experiment lasted for 42 d. On d 21 and 42, the broilers were weighed, and feed consumption was recorded by replication. Average daily weight gain, average daily feed intake, and the ratio of feed to gain were calculated.

### Sample collection and determination of the organ index

At 7, 14, 21, and 28 d of the feeding experiment, one bird from each replicate was randomly selected, and 5 mL of blood from each broiler was collected from the brachial vein into an aseptic glass tube. The blood samples were obliquely placed in a 37 °C environment for 30 min, and then serum samples were separated by centrifugation at 3,500×*g* for 15 min at 4 °C. The supernatant was dispensed into 1.5-mL centrifuge tubes and stored at − 80 °C for further hemagglutination inhibition tests.

Moreover, tissue and serum samples were also respectively gathered at 21 and 42 d of age. For each sampling, one bird from each replicate, with a body weight that was typically close to the average body weight of the broilers from the same replication, was selected and weighed after fasting for 12 h. Blood samples were collected, and serum samples were prepared, followed by euthanasia by exsanguination after intravenous administration of 3% sodium pentobarbital (25 mg/kg body weight; Sigma, USA) and immediate dissection. All efforts were made to minimize animal suffering. First, immune organs (liver, thymus, spleen, and bursa) were collected and weighed immediately. Organ indices were expressed relative to body weight (g of organ/kg of body weight). Then, by removing the contamination of intestinal contents, the middle complete duodenal, jejunal, and ileal segments with lengths of 3 cm were collected and fixed in 10% buffered formalin for at least 24 h for further histological processing and immunohistochemical analysis. Next, the duodenal, jejunal, and ileal mucosa and the caecal content samples were collected into 2-mL Eppendorf tubes and frozen immediately in liquid nitrogen. After collecting all the samples, the duodenal, jejunal, and ileal segments were stored at 4 °C, and the other samples were stored at − 80 °C until analysis.

### Hemagglutination inhibition test

At 3 d of age, the broilers received an intramuscular injection with vaccine (H5N1 Re-5 + H9N2 Re-2 types bivalent inactivated vaccine, Harvak biotechnology company, Harbin, China) for H5 and H9 types of Avian influenza. Then, the collected serum samples from 7, 14, 21, and 28 days old broilers were used for the hemagglutination inhibition test. Hemagglutination inhibition test procedures were performed according to the World Organization of Animal Health manual (OIE, 2004) for the detection of H5 and H9 antibodies. Four hemagglutination units of homologous antigen (HI standard antigen) were used, and the HI antibody titers were expressed as a reciprocal of the highest serum dilution that had complete inhibition of hemagglutination [26].

### Determination of the serum IgG concentration

The serum IgG content was measured using ELISA kits (Shanghai YuanMu Biological Technology Co. Ltd., Shanghai, China) for chicken following the manufacturer’s procedure.

### Determination of intestinal morphology

The middle complete duodenal, jejunal, and ileal segments, which were fixed in 10% buffered formalin, were used for analysis of intestinal morphology. After fixation, the samples were dehydrated and cleared. Then, intestinal samples were cut and inserted into cassettes, which were embedded in liquid paraffin. Next, 5-μm paraffin sections were cut using a microtome and stained with hematoxylin-eosin. Villus height and crypt depth were determined using a phase contrast microscope [27].

### Measurement of intestinal secreted immunoglobulin a (sIgA) by immunohistochemical analysis

Immunohistochemistry analysis was conducted to detect the intestinal sIgA content in the CON, AGP, and AP2 (also named ABP) groups according to a previous study [28]. In brief, the duodenum, jejunum, and ileum were fixed in 10% buffered formalin for 24 h at room temperature and embedded in paraffin. The abundance of sIgA was assessed in 3-mm paraffin embedded slides after the sections were dewaxed in xylene, rehydrated in an ascending ethanol series and pre-treated in a microwave oven (two cycles for 5 min each at 780 W, in EDTA buffer, pH 8.0). Endogenous biotin and non-specific signals were blocked with the appropriate reagents. For immunohistochemistry, the treated slides were incubated with primary antibodies for 2 h at room temperature in a humidified chamber, washed in PBS, and visualized with biotinylated secondary antibodies followed by incubation with HRP-conjugated streptavidin for 30 min (R&D Systems, London, UK). For each sample, the average integral optical density (mean of iod) from at least 10 fields of view were analyzed with image analysis software (Image-Pro Plus 6.0, Maryland, USA).

### RNA isolation of duodenal, jejunal, and ileal mucous membran 

Total RNA from duodenal, jejunal, and ileal mucous membrane samples from 21-day-old and 42-day-old broilers from the CON, AGP, and AP2 (ABP) groups were extracted using TRIzol reagent (Invitrogen, CA, USA). Specifically, DNaseI was used during the RNA isolation process to avoid contamination with genomic DNA. The quantity and purity of total RNA were analyzed using a NanoDrop® ND-1000 spectrophotometer (Thermo Scientific, MA, USA), and the RNA integrity was assessed by gel electrophoresis. Only RNA samples that had an OD260/280 > 1.8, OD260/230 > 2.0 and had good integrity were used for further qRT-PCR.

### Microbial DNA extraction

A total of fifteen caecal content samples (5 of 7 replications randomly selected from each selected group) from 21-day-old and 42-day-old broilers of 3 different treatments (CON, AGP, and AP2 groups) were used for DNA extraction using the QIAamp DNA Stool Mini Kit (Qiagen, German) according to the manufacturer instructions. DNA Samples were measured on a Nanodrop ND-1000 spectrophotometer (Thermo Scientific, USA) to assess DNA quantity and then stored at − 80 °C until sequencing analysis.

### Quantitative real-time PCR (qRT-PCR) analysis

ZApproximately 1 μg of total RNA from the intestinal mucous membrane was reverse-transcribed using the PrimeScript™ RT reagent Kit with gDNA eraser (Takara, Dalian, China). qRT-PCR was performed using SYBR® Green PCR Master Mix (Takara, Dalian, China). A 20-μL PCR mixture was quickly prepared. Primers for *β-actin* (internal control genes) and the tested mRNAs were designed using Primer-BLAST (http://www.ncbi.nlm.nih.gov/tools/primer-blast/) and are listed in Table S4. Briefly, the tested mRNAs were genes involved in nutrient transport: *SGLT1*, *GLUT2*, *rBAT*, *y *^*+*^*LAT2*, and *CAT1*. PCR was conducted in an iCycler iQ5 multicolor real-time PCR detection system (Bio-Rad Laboratories) programmed as follows: 95 °C for 10 min; 40 cycles of 95 °C for 10 s, 60 °C for 30 s, and 72 °C for 30 s; and 72 °C for 5 min. All samples were examined in triplicate. All data were analyzed using the 2^−ΔΔCt^ method [29].

Furthermore, the populations of total bacteria, *Campylobacter jejuni*, *Salmonella gallinarum*, *Escherichia coli*, and *Bifidobacterium bifidum* in the caecum were determined by SYBR green-based absolute qPCR. The bacterial-specific primer sets are shown in Table S5. The qPCR plasmid standard was prepared according to the method described in a previous study [30]. Subsequently, the concentration of the plasmid standards was diluted to 30 ng/μL and subjected to a series of ten-fold dilutions (10^− 1^ –10^− 6^) to obtain the standard curve. The copy number of the diluted plasmid standard was calculated as follows: copy number/μL = [N_A_ × C (ng/μL) × 10^− 9^]/[660 (Da/bp) × L_DNA_(bp)], where N_A_ = 6.02 × 10^23^; C (ng/μL) = plasmid DNA concentration (20 ng/μL); L_DNA_(bp) = length of plasmid DNA (pMD-19 T vector + target fragment). The standard curve of the bacteria was calculated based on the copy number and Ct values.

### The 16S rRNA gene amplification of the V3 + V4 region, sequencing, and bioinformatics analysis

The 16S rRNA gene amplicons were used to determine the diversity and structural comparisons of the bacterial species in caecal samples using Illumina MiSeq sequencing at LC Bio Tech Co., Ltd., Zhejiang, China. The V3 + V4 hypervariable region of the 16S rRNA gene was PCR amplified from microbial genomic DNA harvested from caecal content samples using forward primer 338F (5′-ACTCCTACGGGAGGCAGCAG-3′) and reverse primer 806 R (5′- GGACTACNNGGGTATCTAAT-3′) [31].

Paired-end reads were assigned to samples based on their unique barcode, and samples were truncated by cutting off the barcode and primer sequences. Paired-end reads were merged using FLASH [32]. Quality filtering of raw tags was performed using specific filtering conditions to obtain high-quality clean tags with FastQC. Verseach (v2.3.4) [33] was used to filter chimeric sequences and assign samples with ≥97% sequence similarity to the same operational taxonomic units (OTUs). The representative sequence of each cluster was selected to represent the operational taxonomy units (OTUs) [34]. Representative sequences were chosen for each OTU, and taxonomic data were assigned to each representative sequence using the Ribosomal Database Project (v11.5) [35] and NCBI classifier. OTU abundance data were normalized using a standard sequence number corresponding to the sample with the least number of sequences. These indices were calculated for our samples using QIIME software (Version 1.9.0) [36]. The taxon abundance for each sample was determined according to the phylum, class, order, family, and genus. The alpha diversity was identified by studying the observed species, Chao1, Goods_coverage, and Shannon and Simpson indices. The microbiota were compared for beta diversity using the distance matrices generated from weighted UniFrac analysis, principal coordinated analysis (PCoA) and ANOMIS analysis. The Mann-Whitney U test (*P* < 0.05) was used to identify differences between the samples. Moreover, LEfSe analysis was performed to estimate the effect size of species that contributed to the differences between samples. The threshold of the LDA score was set at a default value of 3.0 and *P* value less than 0.05. Correlations between variables were tested by the Pearson correlation test, and RDA analyses were performed using R packages.

### Statistical analysis

The analysis was performed by One-way ANOVA using SPSS 21.0 software with replicates as experimental units and differences considered to be statistically significant at *P* < 0.05. Significant differences at the 0.05 level due to treatments were distinguished by Duncan’s multiple range tests. Furthermore, to clarify the best inclusion levels of Api-PR19, curvilinear regression analysis was further performed based on the supplementation concentration of Api-PR19 and significantly altered growth performance (the ratio of feed to weight of broilers from 1 to 42 d) using SPSS 21.0 software.

### Data availability

The sequence data were deposited and are available in the Sequence Read Archive (SRA) of NCBI under accession project number PRJNA578221.

## Results

### Apidaecin Api-PR19 exhibits good antimicrobial activity*in vitro*

**Fig. 1:**
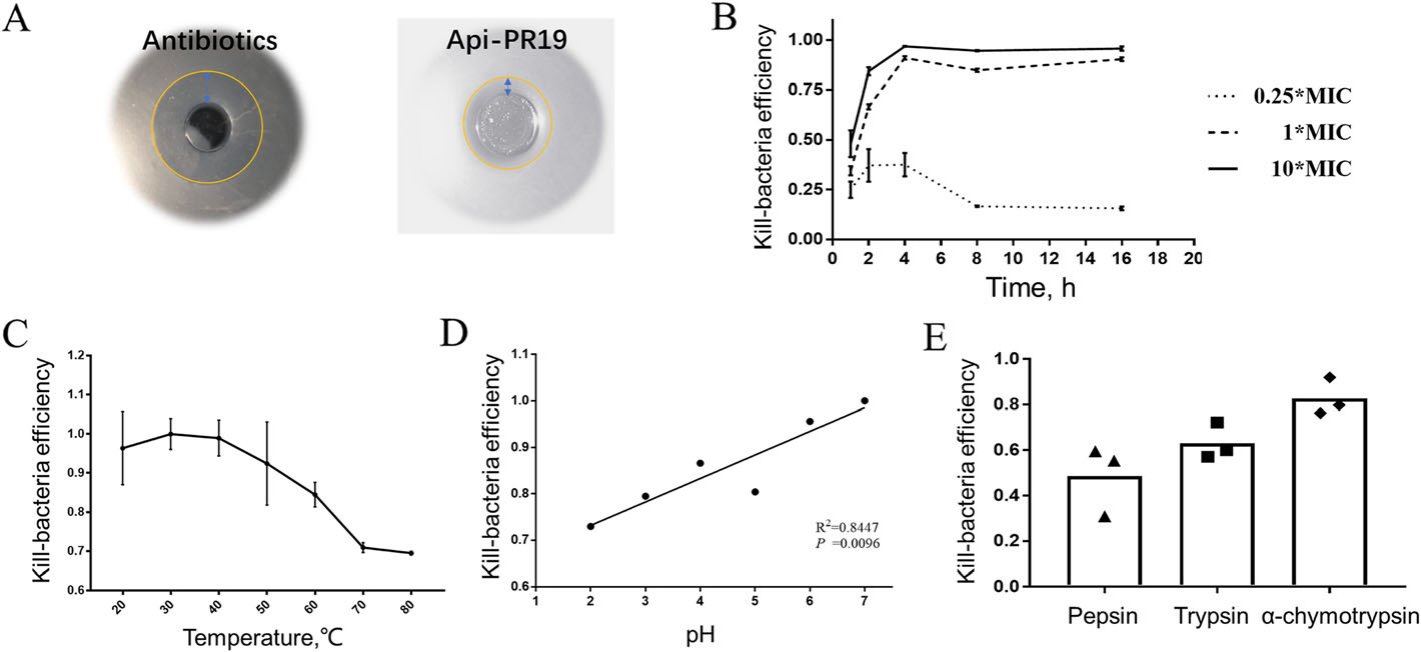
Antimicrobial activity tests and stress resistance tests for apidaecin Api-PR19 *in vitro*. (**a**) Zone of inhibition of Api-PR19 and enramycin (1 mmol/L concentration) using the Oxford Cup method. (**b**) Bacterial killing efficiency of Api-PR19. For Api-PR19, different shapes are used to indicate three concentrations: 10×(continuous line), 1×(intermittent line) and 0.25×(circle dot). (**c-e**) Effect of different temperature, pH, and protease on bacterial killing efficiency of Api-PR19. All data are represented as the mean ± SD

By using the *in vitro* minimum antimicrobial concentration (MIC) assay, the MIC of apidaecin Api-PR19 for inhibiting *Escherichia coli*, *Salmonella typhimurium*, *Staphylococcus aureus*, *Helicobacter pylori*, and *Pasteurella* growth was 0.2, 0.4, 1.6, 0.8, and 0.1 μg/mL, respectively (Table 1). Similar results were also obtained by the Oxford Cup methods, which also indicated that the bacteriostatic effect of apidaecin Api-PR19 was less than the antibiotics (Fig. 1a). By further using *Escherichia coli* as the indicator, the increase in the apidaecin Api-PR19 concentration could significantly increase the bactericidal activity. Moreover, the bactericidal activity could significantly increase with the increased time up to 4 h, followed by a decrease after 4 h and then maintenance after 8 h (Fig. 1b). Overall, apidaecin Api-PR19 exhibited antimicrobial activity, demonstrating its potential direct use as a replacement for antibiotics.

**Table 1:** The minimum antimicrobial concentration (MIC) of Api-PR19 and the MIC of the previous reported different antibiotics

Bacteria	MIC, (μg/mL)/ (μmol /mL)	
	Api-PR19	Antibiotics
*Escherichia coli* ATCC25922	0.2 / 8.5 × 10^− 5^	0.015 / 4.53 × 10^–5^ [37] (Ciprofloxacin)
Salmonella typhimurium ATCC14028	0.4 / 1.72 × 10^− 4^	0.0975 / 2.42 × 10^–4^ [38] (Ampicillin)
Pasteurella ATCC19427	0.1 / 4.29 × 10^− 5^	0.012 /1.80 × 10^–5^ [39] (Cefoperazone)
Helicobacter Pylori ATCC43504	0.8/ 3.43 × 10^− 4^	0.0312 / 4.17 × 10^–^ 5 [40] (Clarithromycin)
*Staphylococcus aureus* ATCC25923	1.6/ 6.86 × 10^− 4^	0.125 / 2.94 × 10^–4^ [41] (Clindamycin)

### Apidaecin Api-PR19 has resistance ability to high temperature, low pH, and several proteases *in vitro*

The effects of different factors, including enzymes, pH and temperature, on Apidaecin Api-PR19 stability were evaluated (Fig. 1c-e). With the increase in temperature, the antimicrobial activity of Api-PR19 significantly decreased, but it also maintain 80% of its antimicrobial activity even when the temperature was raised to 80 °C. Specifically, the antimicrobial activity remained highest from 30 to 40 °C, which is the body temperature range in chickens (Fig. 1c). Moreover, with the decrease in pH, the antimicrobial activity of Api-PR19 also gently decreased and maintained activity of more than 72% even when the pH decreased to 2 (Fig. 1d). Specifically, the gastric and intestinal pH of broilers ranged from 2.6 to 6.4, among which the gizzard has the lowest pH. Thus, the antimicrobial activity of Api-PR19 could be mostly retained during passage through the gastrointestinal tract of broilers. Furthermore, after treatment with pepsin (3,000–3,500 NFU), trypsin (250 NFU), and α-chymotrypsin (1,200 U), Api-PR19 could also retain 52%, 63%, and 82%, respectively, of the antimicrobial activity (Fig. 1e). Hence, by considering the gastrointestinal conditions in broilers, although the loss of activity due to enzyme activity appeared significant, Api-PR19 could also act in the gut of broilers to exert its antimicrobial effects.

### Effects of apidaecin Api-PR19 on the growth performance and immune function of broilers

During the 42-day feeding experiment, there were no significant differences in average daily feed intake (ADFI) and average daily weight gain (ADG) among the different groups, including the CON, AGP, AP1, AP2, and AP3 group (Table 2). However, the ratio of feed to weight (also known as the feed conversion ratio, FCR) of broilers in the AP2 and AP3 groups during the 1–21 d and 1–42 d periods were significantly decreased when compared with the CON group (Table 2). Moreover, when compared with the CON group, the FCR of broilers in the AGP group during the 1–42 d periods was also significantly decreased.

**Table 2:** Effects of Api-PR19 and antibiotic on growing performance, immune organ index, and immune function of 21-day-old and 42-day-old broiler chickens

Item	Period/age	Indices	CON	AGP	Api-PR19, mg/kg			SEM	*P*-value
					100	200	300		
Growth performance	1–21 d	ADFI, g	54.49	54.28	54.72	54.72	54.13	0.439	0.992
		ADG, g	39.09	39.74	39.99	40.57	39.85	0.325	0.735
		FCR	1.394^a^	1.366^ab^	1.367^ab^	1.349^b^	1.359^b^	0.005	0.041
	22–42 d	ADFI, g	147.45	152.54	146.29	151.25	150.32	1.815	0.819
		ADG, g	72.58	76.12	71.13	77.28	75.31	1.157	0.442
		FCR	2.033	2.006	2.065	1.959	1.998	0.068	0.070
	1–42 d	ADFI, g	97.16	101.06	97.11	100.21	98.86	1.161	0.786
		ADG, g	53.03	56.62	53.41	57.20	55.65	0.729	0.262
		FCR	1.832^a^	1.783b^c^	1.820^b^	1.753^c^	1.775^c^	0.008	0.005
Immune organ development	21 d	Thymus index	2.20	2.02	2.22	2.13	2.22	0.081	0.555
		Spleen index	0.88	0.78	0.83	0.67	0.70	0.033	0.338
		Bursa index	2.37^a^	1.60^c^	1.77^b^	1.72^b^	2.15^a^	0.772	0.025
	42 d	Thymus index	3.24	3.01	3.44	2.70	2.79	0.150	0.519
		Spleen index	0.95	0.90	0.93	0.88	0.83	0.028	0.130
		Bursa index	0.48	0.45	0.52	0.38	0.35	0.031	0.387
IgG	21 d	Serum IgG	386.14	462.21	355.86	340.43	304.71	20.264	0.156
	42 d	Serum IgG	636.86	524.71	732.33	430.90	731.38	44.642	0.158
H_5_ & H_9_ antibody level (log_2_)	7 d	H_5_	8.29	7.71	8.14	8.28	8.71	0.771	0.193
	14 d		6.43	5.86	5.71	6.00	6.00	0.797	0.566
	21 d		4.28	3.85	5.00	4.43	4.43	0.743	0.059
	28 d		4.29	3.43	4.14	3.14	3.57	1.269	0.416
	7 d	H_9_	9.57^a^	8.00^b^	9.57^a^	9.43^a^	10.29^a^	1.374	0.024
	14 d		7.29	6.86	6.71	6.43	6.43	1.121	0.612
	21 d		4.43	4.00	4.14	4.43	4.57	0.900	0.775
	28 d		3.57	3.71	2.86	3.29	4.00	1.091	0.363

Note: *ADFI* average daily feed intake; *ADG* average daily weight gain; *FCR* ratio of feed intake to weight gain. ^a-b^ within a row with different superscripts means significantly difference

One of the potential beneficial roles of AGP in promoting the growth performance of broilers is by defending against pathogenic bacteria and improving immune function [12]. The present study only found that supplementation of AGP and apidaecin Api-PR19 could both decrease the bursa index of 21-day-old broilers (Table 2). Moreover, no difference was identified among the 5 treatments when measuring the serum IgG level (Table 2). However, the decrease in antibody levels against H9 avian influenza in the AGP group indicated a decrease in adaptive immune function and increased potential health risk of broilers when using antibiotics (Table 2). Overall, both antibiotics and apidaecin Api-PR19 could be beneficial to growth performance when compared to the CON group, but the antibiotics could increase potential health risks.

Furthermore, based on the curvilinear regression analysis, a regression equation was established based on the supplementation amount of Api-PR19 and FCR of broilers during the 1–42 d periods (Table 3). As a result, the optimum levels of Api-PR19 were 222.9 mg/kg for the 1–42 d period.

**Table 3:** Optimum Api-PR19 levels in broiler chickens on regression equation (mg/kg)

Regression equation	R^2^	*P*-value	Optimum levels, mg/kg
y = 2.321–0.003x + 6.729E-6 × 2	0.325	0.029	222.9

Note: *x* represents the optimum levels of Api-PR19, *y* represents the ratio of feed intake to weight gain (FCR) of the broilers

### Beneficial effects of apidaecin Api-PR19 on the intestinal morphology, absorption function, and healthy condition of broilers

**Fig. 2:**
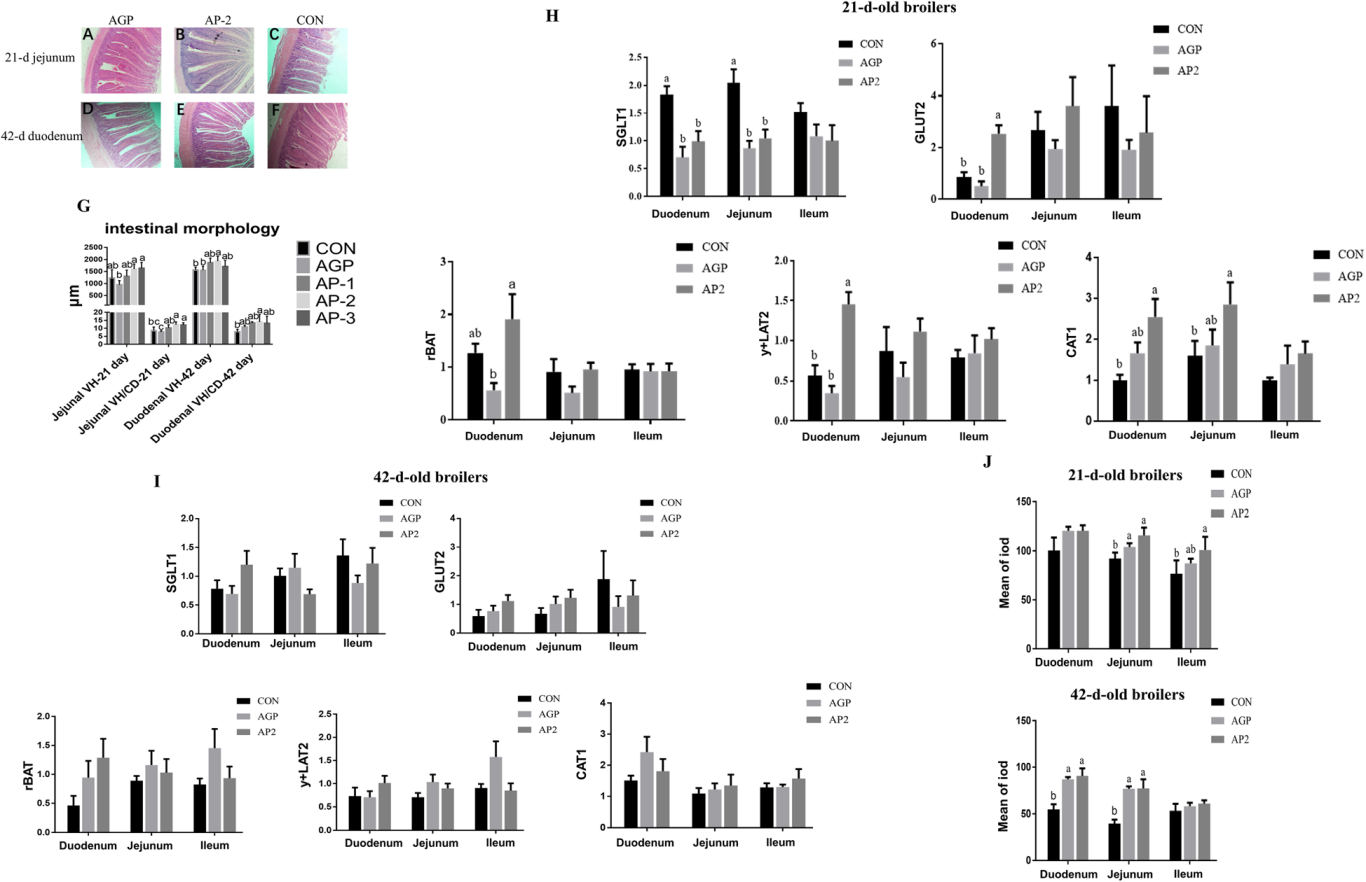
Effect of Api-PR19 and enramycin on the intestinal morphology of 21-day-old and 42-day-old broilers (**a-g**), on the relative mRNA expression of glucose transporters (SGLT1, GLUT2) and amino acid transporters (rBAT, y^+^LAT2, CAT1) of 21-day-old (**h**) and 42-day-old broilers (**i**), and on the intestinal sIgA content of 21-day-old and 42-day-old broilers (**j**). Note: CON indicates control group; AP1, AP2, and AP3 indicate the groups with 100, 200, and 300 Api-PR19; AGP indicates enramycin (antibiotic) group. The iod in (**j**) represent the integral optical density measured by Image-Pro Plus 6.0 software

The gut is the most important site for nutrient absorption and resistance to pathogens due to its immune function, and the intestinal conditions of broilers were further measured to identify the cause of improved growth and healthy condition in broilers fed with apidaecin. The intestinal morphology analyses showed that apidaecin Api-PR19 significantly increased the jejunal villus height and ratio of the villus height to crypt depth of 21-day-old broilers, as well as the duodenal villus height and ratio of villus height to crypt depth of 42-day-old broilers, when compared with the CON and AGP group. When compared with the apidaecin Api-PR19 supplementation groups, the supplementation of AGP disrupted the jejunal villus of 21-day-old broilers and duodenal villus of 42-day-old broilers (Fig. 2a-g, Table S6).

The optimum levels of apidaecin Api-PR19 based on the FCR of broilers was 222.9 mg/kg for the 1–42 d period, which was closer to 200 mg/kg (AP2 group). Hence, to illuminate how Api-PR19 served to promote the growth performance of broilers, the AP2 group was selected as the representative group among the 3 apidaecin groups to identify the differential roles of Api-PR19 and AGP in regulating intestinal absorption and immune function, as well as the gut microbiota. For intestinal absorption, the mRNA expression levels of CAT1, rBAT, y^+^LAT2, GLUT2, and SGLT1 in the duodenum, jejunum, and ileum of 21-day-old and 42-day-old broilers were determined (Fig. 2h and i). At 21 d of age, the expression level of SGLT1 significantly decreased in the AGP and apidaecin groups compared with the CON group. However, supplementation with apidaecin significantly increased duodenal GLUT2, rBAT, y^+^LAT2, and CAT1 expression as well as jejunal CAT1 expression compared with the CON and AGP group. Furthermore, intestinal sIgA contents were also measured to assess intestinal immune function. Both AGP and apidaecin supplementation could increase jejunal sIgA content in 21-day-old broilers and increase duodenal and jejunal sIgA contents in 42-day-old broilers (Fig. 2j). Overall, the beneficial effects of apidaecin Api-PR19 were induced by the improved intestinal absorption and immune function, while the AGPs enhanced intestinal immune function but harmed the intestinal morphology and absorption function.

### Apidaecin Api-PR19 can maintain gut microbial homeostasis with reduced pathogen abundance

**Fig. 3:**
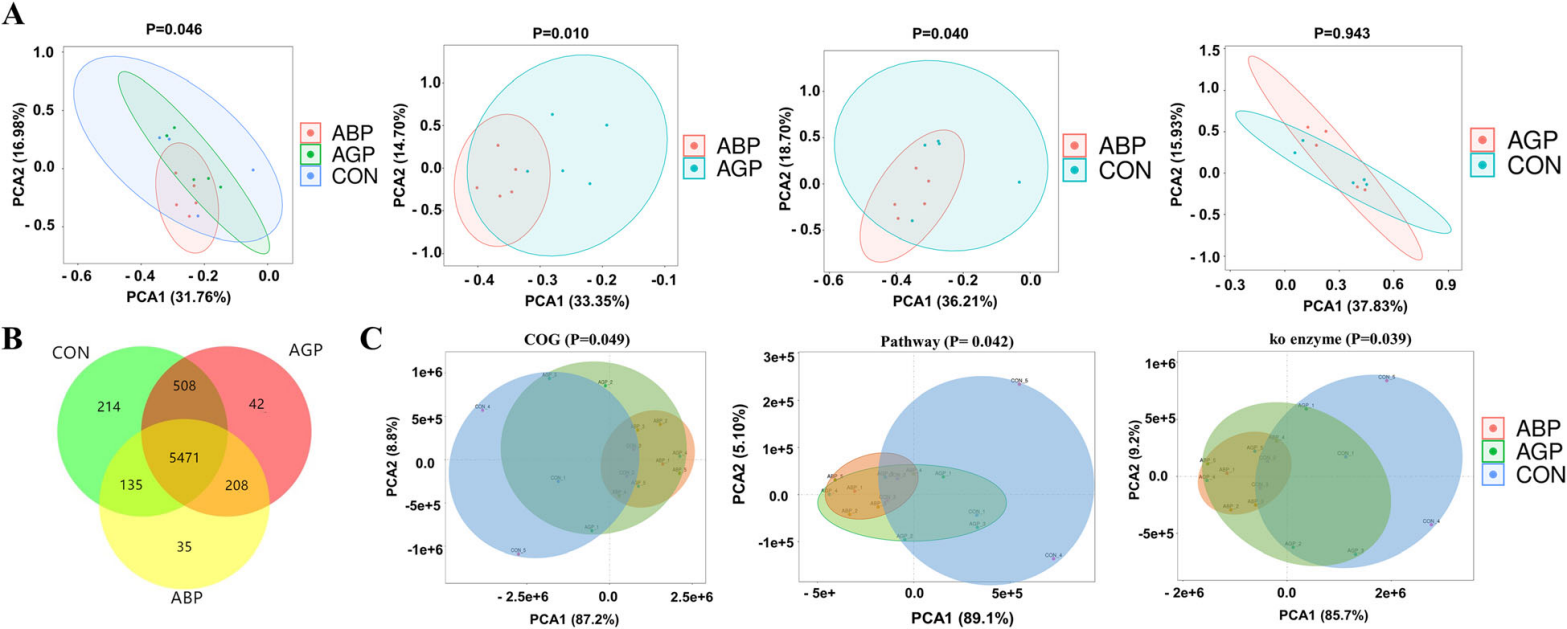
Both Api-PR19 and antibiotic could alter the gut microbiota of broilers. (**a**) PCA analyses identified differences in gut microbiota among CON, AGP, and ABP. (**b**) Venn diagram analyses based on the identified OTUs in CON, AGP, and ABP. (**c-e**) PCA analyses identified differences in microbial function among CON, AGP, and ABP based on COG enzyme, pathway, and ko enzyme analyses. Note: CON indicates control group; ABP indicates Api-PR19 group; AGP indicates enramycin (antibiotic) group

When determining the different roles of antibiotics and apidaecin, their effects on the gut microbial population were further detected. According to the qRT-PCR results, we found that both AGP and apidaecin Api-PR19 could significantly decrease the population of *Escherichia coli* and *Campylobacter jejuni* (Table 4). However, antibiotics significantly decreased the abundance of total bacteria, while the apidaecin resulted in no significant decrease in the abundance of total bacteria, which indicated that apidaecin might not kill other bacteria under the effects of antibiotics (Table 4). Further 16S rRNA gene sequencing results indicated that supplementation with both antibiotics and apidaecin had no significant influence on the diversity of the microbiota, including the Chao1, Observed-species, Shannon, and Simpson indices (Table 4). However, the Venn diagram indicated that the identified OTUs in the different groups were not the same, with 214, 42, and 35 unique OTUs in the CON, AGP, and ABP groups, respectively (Fig. 3b). Furthermore, a significant distinction between the ABP and AGP groups (*P* = 0.01) or between the ABP and CON groups (*P* = 0.04) was identified, which implied that the intestinal microbiota had been significantly altered by apidaecin and might help Api-PR19 combat conditioned pathogens and maintain intestinal health (Fig. 3a). Moreover, the PCA analyses based on the COG, pathway and ko enzyme analyses all identified that bacterial functions were separately clustered by treatment (Fig. 3c, d, e). Specifically, the AGP and ABP groups clustered better than CON, which indicated that the antibiotics and apidaecin had selective effects on the microbiota (Fig. 3c-e). Moreover, the ABP group clustered better than AGP group, representing the intestinal microbial structure shift in different directions in response to antibiotic treatment, which might be induced by antibiotic resistance. Overall, both antibiotics and apidaecin could combat conditioned pathogens, but apidaecin had a reduced effect on other bacteria, which resulted from the non-significant change in total bacteria. Herein, the different roles of antibiotics and apidaecin in regulating the microbiota were worthy of further study.

**Table 4:** Effect of Api-PR19 and antibiotic on the conditioned pathogen abundance and the microbiota of broilers’ gut

	Item	CON	AGP	AP2 (200 mg/kg Api-PR19)	SEM	*P*-value
21 d	Total bacteria	16.47	16.63	16.80	0.15	0.704
	*Escherichia coli*	12.74	12.51	12.94	0.23	0.338
	*Bifidobacterium bifidum*	13.20	13.85	13.44	0.18	0.391
	*Salmonella gallinarum*	13.17	13.42	13.40	0.24	0.925
	*Campylobacter jejuni*	11.16	11.84	11.65	0.19	0.419
42 d	Total bacteria	14.93^a^	14.04^b^	14.48^ab^	0.14	0.019
	*Escherichia coli*	9.92^a^	8.54^b^	8.46^b^	0.25	0.011
	*Bifidobacterium bifidum*	13.36	12.62	12.96	0.15	0.138
	*Salmonella gallinarum*	12.98	12.58	12.55	0.11	0.256
	*Campylobacter jejuni*	11.84^a^	10.83^b^	11.49^ab^	0.18	0.043
42 d α-diversity	Observed species	2947.60	3124.60	2955.80	104.89	0.767
	Shannon	8.20	8.34	8.35	0.13	0.888
	Simpson	0.972	0.974	0.980	0.005	0.801
	Chao1	3928.21	4158.35	3883.47	142.29	0.730
	Goods coverage	0.976	0.974	0.978	0.0016	0.641

Note: ^a-b^ within a row with different superscripts means significantly difference

### Apidaecin Api-PR19 and antibiotics induce differential alterations of the gut microbiota

The differential bacteria between groups, including ABP vs. AGP, ABP vs. CON, and AGP vs. CON, were analyzed using the Mann-Whitney U test at different levels (Table 5). At the phylum level, a significant increase in Proteobacteria was identified in the AGP group compared with the ABP group. At the class level, ABP supplementation could significantly decrease the Deltaproteobacteria but increase the Bacteria-unclassified compared with the AGP group. At the family level, compared with the CON group, ABP supplementation could significantly increase the Prevotellaceae, while AGP supplementation could significantly increase the Bacillaceae_2 and significantly decrease the Christensenellaceae. Moreover, compared with the AGP group, ABP supplementation could significantly increase the Eubacteriaceae, Christensenellaceae, Bacteria_unclassified, and Gracilibacteraceae while decreasing the Bacillaceae-2 and Acidaminococcaceae. At the genus level, compared with the CON group, AGP supplementation significantly increased the *Bacillaceae_2_unclassified* and *Bacillus* but decreased the *Peptococcus*, while ABP supplementation significantly decreased the *Holdemania*, *Tyzzerella* and *Prevotella*. Moreover, compared with the AGP group, ABP supplementation significantly increased the *Christensenella*, *Eubacterium*, *Bacteria*_*unclassified*, and *Gracilibacter* but decreased the *Bacillaceae_2_unclassified*, *Bacillus*, *Faecalibacterium*, and *Tyzzerella*. At the species level, when compared with the CON group, AGP supplementation could significantly increase *Clostridium_sp._enrichment_culture_clone_06–1,235,251-143*, *Bacillaceae_2_unclassified*, and *s__Bacillus_sp._CW27-B01* and decrease *Parasutterella_secunda*, *Sporobacter_termitidis* and *uncultured_Peptococcus_sp*; ABP supplementation could significantly decrease *Anaeromassilibacillus_sp._Marseille-P4683*, *Prevotella_lascolaii*, *Holdemania_filiformis*, *Clostridium_colinum*, and *Helicobacter_cf._pullorum* but increase *Clostridium_asparagiforme* and *uncultured_Christensenella_sp*. Moreover, compared with the AGP group, ABP supplementation could significantly increase the *uncultured_Christensenella_sp*., *uncultured_Eubacterium_sp*., *Alistipes_unclassified*, *Bacteria_unclassified*, *uncultured_Gracilibacter_sp*. and decrease *Bacillaceae_2_unclassified*, *Bacillus*_sp._*CW27-B01*, *Clostridium_colinum*, *Faecalibacterium*_*prausnitzii*, and *Anaeromassilibacillus*_*sp*._*Marseille-P4683*. Furthermore, similar results but less differential bacteria were also identified using linear discriminant analysis (LDA) and effect size (LEfSe) analysis (Fig. S1A-C).

**Table 5:** The differential bacteria among CON, AGP, and ABP identified using the Mann-Whitney U test

Bacteria	ABP	AGP	log_2_FC	Wilcox.test. *P*-value
ABP vs. AGP				
Phylum				
Proteobacteria	0.5614	1.4075	− 1.33	0.0472
Class				
Deltaproteobacteria	0.3681	0.7903	−1.1	0.0472
Family				
Bacillaceae 2	0	0.0147	-Inf	0.0186
Eubacteriaceae	4.1316	1.5342	1.43	0.0283
Christensenellaceae	0.1028	0.0456	1.17	0.0283
Acidaminococcaceae	0.0866	0.2892	−1.74	0.0472
Gracilibacteraceae	0.0062	0.0027	1.2	0.0472
Genus				
*Christensenella*	0.083	0.0272	1.61	0.009
*Bacillaceae 2 unclassified*	0	0.0147	-Inf	0.0186
*Bacillus*	0	0.0038	-Inf	0.0186
*Faecalibacterium*	3.2023	9.9469	−1.64	0.0283
*Eubacterium*	4.1152	1.525	1.43	0.0283
*Tyzzerella*	0.0005	0.0069	−3.79	0.0343
*Gracilibacter*	0.0062	0.0027	1.2	0.0472
Species				
*Uncultured Christensenella* sp.	0.0547	0.0097	2.5	0.0163
*Bacillaceae 2 unclassified*	0	0.0147	-Inf	0.0186
*Bacillus* sp. CW27-B01	0	0.0038	-Inf	0.0186
*Anaeromassilibacillus* sp. Marseille-P4683	0.0004	0.0037	−3.21	0.0236
*Uncultured Eubacterium* sp.	4.1146	1.5238	1.43	0.0283
*Clostridium colinum*	0.0005	0.0069	−3.79	0.0343
*Faecalibacterium prausnitzii*	1.82	5.116	−1.49	0.0472
*Alistipes unclassified*	3.8294	1.5695	1.29	0.0472
*Uncultured Gracilibacter* sp.	0.0062	0.0027	1.2	0.0472
ABP vs. CON				
Family				
Prevotellaceae	0	0.0496	-Inf	0.0186
Genus				
*Holdemania*	0.0189	0.0618	−1.71	0.009
*Tyzzerella*	0.0005	0.0199	−5.31	0.0132
*Prevotella*	0	0.0496	-Inf	0.0186
Species				
*Anaeromassilibacillus* sp. Marseille-P4683	0.0004	0.0084	−4.39	0.0071
*Holdemania filiformis*	0.0189	0.0618	−1.71	0.009
*Clostridium colinum*	0.0005	0.0199	−5.31	0.0132
*Clostridium asparagiforme*	0.0064	0.0007	3.19	0.016
*Prevotella lascolaii*	0	0.0496	-Inf	0.0186
*Helicobacter cf. pullorum*	0	0.0028	-Inf	0.0186
*Uncultured Christensenella* sp.	0.0547	0.0129	2.08	0.0283
AGP vs. CON				
Family				
Bacillaceae 2	0.0147	0	Inf	0.0186
Christensenellaceae	0.0456	0.0714	−0.65	0.0472
Genus				
*Bacillaceae 2 unclassified*	0.0147	0	Inf	0.0186
*Bacillus*	0.0038	0.0001	5.25	0.0343
Species				
*Parasutterella secunda*	0	0.0668	-Inf	0.0186
*Bacillaceae 2 unclassified*	0.0147	0	Inf	0.0186
*Clostridium* sp. enrichment culture clone 06–1,235,251-143	0.0032	0.001	1.68	0.0278
*Sporobacter termitidis*	0.2204	0.8674	−1.98	0.0283
*Bacillus* sp. CW27-B01	0.0038	0.0001	5.25	0.0343
*Uncultured Peptococcus* sp.	0.0056	0.0285	−2.35	0.0472

Note: CON indicates control group; ABP indicates Api-PR19 group; AGP indicates enramycin (antibiotic) group; *-inf* negative infinity; *inf* positive infinity

### The gut microbial community contributes to maintaining intestinal health and promoting growth of broilers and intestinal development

**Fig. 4:**
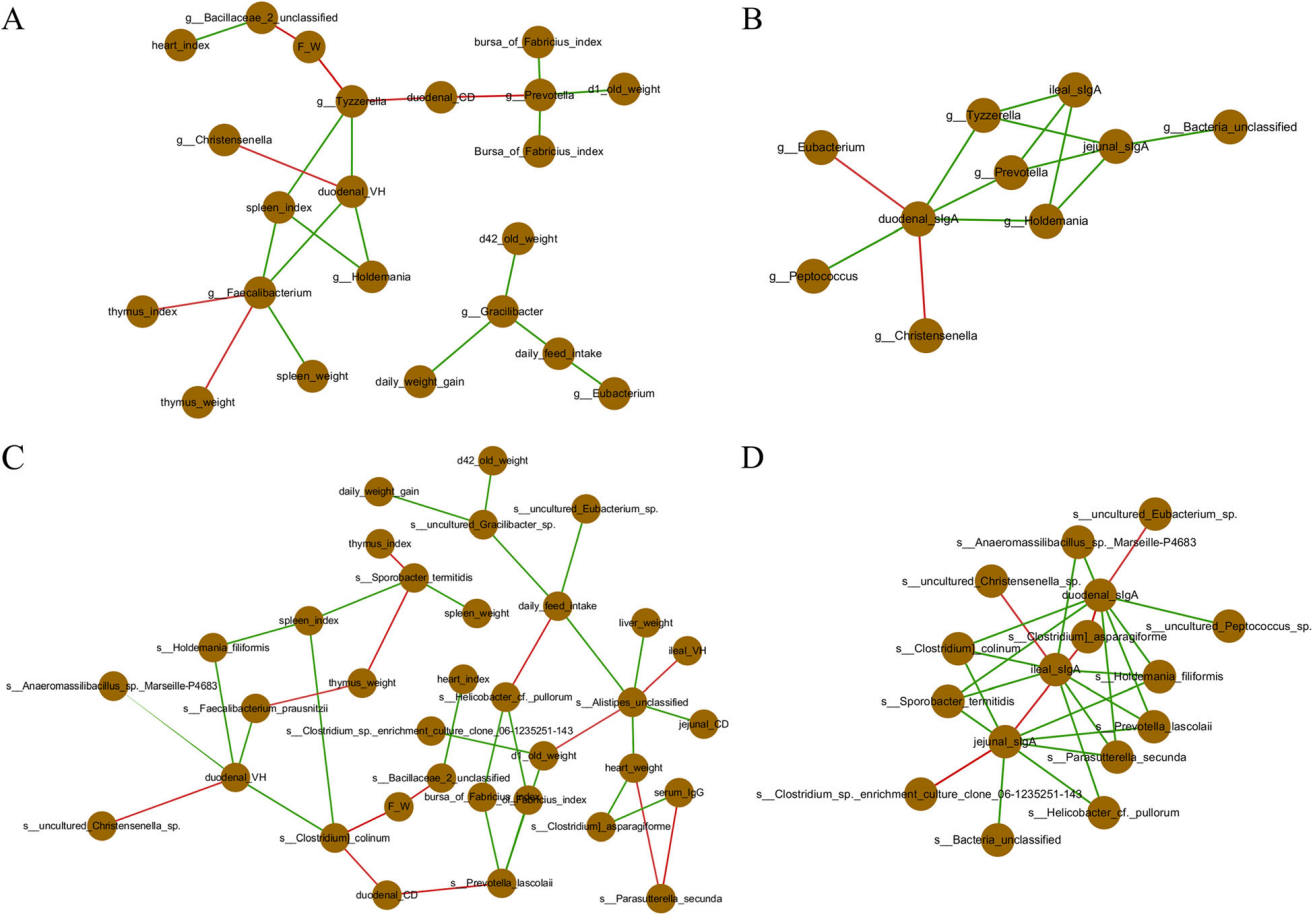
Spearman correlation analyses based on the identified differential genera (**a** and **b**) and species (**c** and **d**) and significantly altered growth performance and intestinal functions. Note: CON indicates control group; ABP indicates Api-PR19 group; AGP indicates enramycin (antibiotic) group. The red line denotes a positive correlation between two points, and the green line denotes a negative correlation between two points

According to the Spearman correlation analyses, we found several genera and species that separately correlated with growth performance and intestinal functions (Fig. 4). For growth performance, we found that the genera of *Gracilibacter*, *Prevotella*, *Eubacterium*, *Tyzzerella*, and *Bacillaceae* and the species of *Bacillaceae_2_unclassified*, *uncultured_Gracilibacter_sp*., *uncultured_Eubacterium_sp*., *Alistipes_unclassified*, *Clostridium_sp._enrichment_culture_clone_06–1,235,251-143* and *Prevotella_lascolaii* were negatively correlated with improved growth performance when considering the increased indices of body weight, daily weight gain, daily feed intake, organ development and decreased ratio of feed to weight index (Fig. 4a). For intestinal functions, the genera of *Tyzzerella*, *Prevotella*, and *Holdemania* were all negatively correlated with the duodenal, jejunal, and ileal sIgA contents (Fig. 4b), while these 3 genera were also negatively correlated with the duodenal morphology (Fig. 4a). Moreover, *Eubacterium* and *Christensenella* were positively correlated with duodenal_sIgA, and Christensenella was positively correlated with duodenal villus height (Fig. 4b). At the species level, *Helicobacter_cf._pullorum*, *Parasutterella_secunda*, *Prevotella_lascolaii*, *Holdemania_filiformis*, *Clostridium_colinum*, and *Sporobacter_termitidis* were negatively correlated with the duodenal, jejunal, and ileal sIgA contents, and *uncultured_Eubacterium_sp*., *Clostridium_sp._enrichment_culture_clone_06–1,235,251-143*, *Clostridium_asparagiforme*, and *uncultured_Christensenella_sp*. were positively correlated with the duodenal, jejunal, and ileal sIgA contents (Fig. 4d). Moreover, the species of *Clostridium_colinum*, *Holdemania_filiformis*, *Anaeromassilibacillus_sp._Marseille-P4683*, *Prevotella_lascolaii*, and *Faecalibacterium_prausnitzii* were negatively correlated with the improved duodenal morphology, which includes an increased villus height and decreased crypt depth, while the species of *uncultured_Christensenella_sp.* were positively correlated with the improved duodenal morphology (Fig. 4c).

### Apidaecin Api-PR19 synergizes with the gut microbial community to combat conditioned pathogens, maintain intestinal health, and promote growth and intestinal development of broilers

**Fig. 5:**
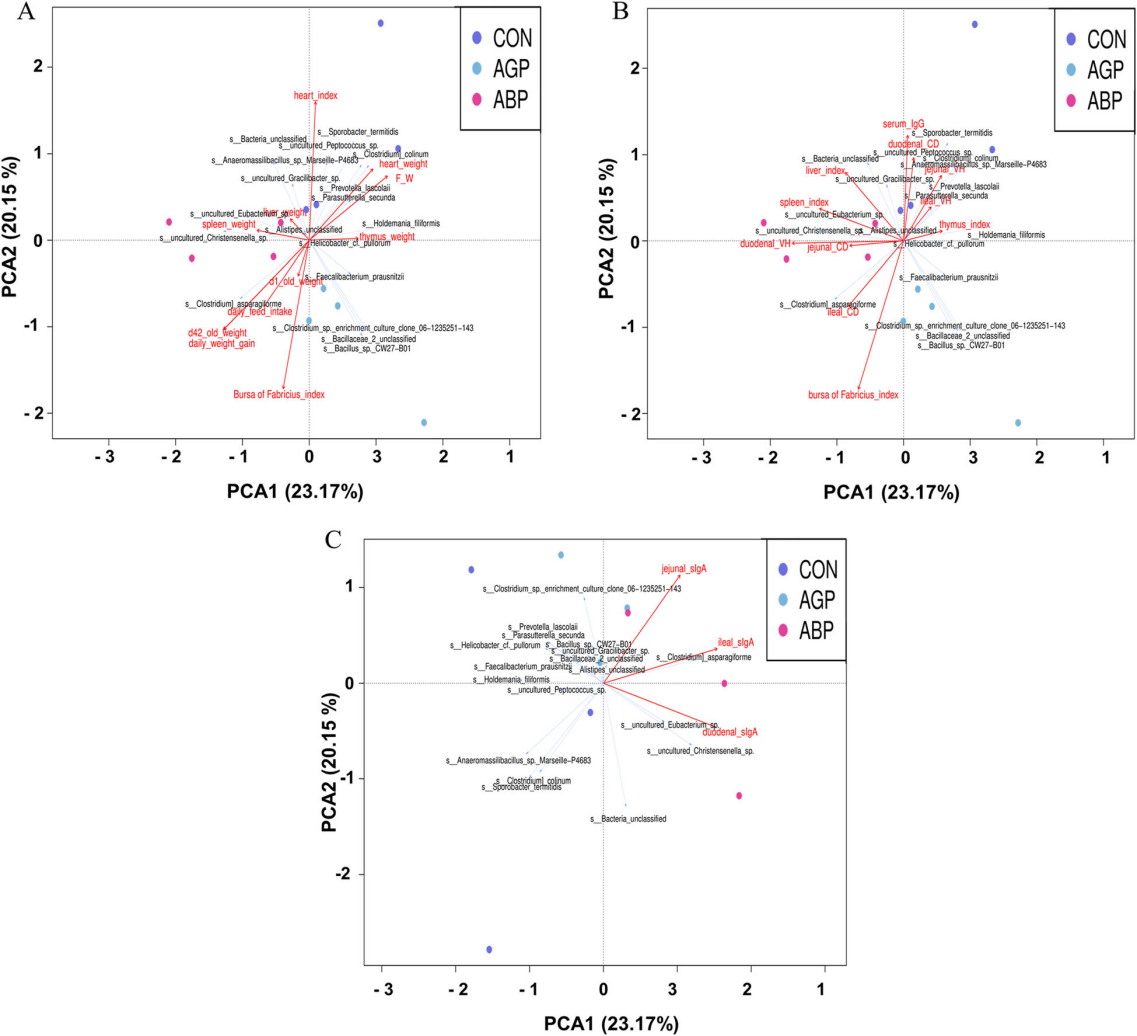
RDA analyses based on the identified differential species and significantly altered growth performance (**a**) and intestinal functions (**b** and **c**) related indices. Note: CON indicates control group; ABP indicates Api-PR19 group; AGP indicates enramycin (antibiotic) group. For the relationship between the differential species and identified altered phenotypes, if the angle between the two lines was less than 90 degrees, a positive correlation was identified; if the angle between the two lines was greater than 90 degrees, a negative correlation was identified. For the relationship between groups and differential species or identified altered phenotypes, if the plot projection of one sample occurred in the positive direction of the extending line of the differential species or the identified altered phenotypes, then the treatment of this sample could increase the abundance of this species or promote this phenotypic change

Furthermore, RDA analyses were performed to identify the relationship among the microbiota, performance, and treatment (Fig. 5). Similar results were obtained for the correlation between differential microbiota and altered growth performance and intestinal functions. The plot projection of the AGP and ABP groups showed a positive direction of the extending line for increased daily feed intake and increased body weight at d 21 and 42, as well as a negative direction of the extending line for the increased ratio of feed intake to weight gain. However, the plot projection for the CON groups showed a negative direction of the extending line for increased daily feed intake and increased body weight of broilers at d 21 and 42, as well as a positive direction of the extending line for the increased ratio of feed intake to weight gain (Fig. 5a). These results showed that the microbiota in the AGP and ABP groups correlated positively with the increased growth performance of broilers, which indicated that supplementation with ABP and AGP was actually beneficial to the growth performance of broilers. We also found that the plot projection of the CON group showed a positive direction of the extending line for the increased jejunal and ideal villus height; the ABP group showed a positive direction of the extending line for increased duodenal villus height. However, the plot projection of the CON group showed a negative direction of the extending lines for increased duodenal, jejunal, and ideal villus height (Fig. 5b). These results indicated that AGP supplementation was harmful to the intestinal morphology. Moreover, the plot projection of the ABP and AGP groups showed a positive direction of the extending lines for increased intestinal sIgA content, while CON displayed a negative direction of the extending line for increased intestinal sIgA content (Fig. 5c). These results indicated that ABP and AGP could increase the sIgA content. Specifically, the ABP group was concentrically distributed near the positive direction of the extending line of the increased abundance of *uncultured_Eubacterium_sp* and *uncultured_Christensenella_sp*, which were meanwhile positively correlated with significantly increased daily feed intake, increased body weight of broilers at days 21 and 42 (Fig. 5a), increased duodenal villus height (Fig. 5b), and increased intestinal sIgA content (Fig. 5c). These results indicated that ABP supplementation could increase the abundance of *uncultured_Eubacterium_sp* and *uncultured_Christensenella_sp*, and these 2 species could play important roles in increasing growth performance and intestinal health.

## Discussion

The apidaecins have special antibacterial mechanisms and are non-toxic to human cells, a prerequisite for their use as a novel antibiotic replacement [42]. According to a previous study, the amino acid composition of apidaecins could determine their antimicrobial activity and stress resistance. For instance, previous studies have demonstrated that N-terminal mutant forms of apidaecin have stronger inhibitory activity toward Gram-negative bacteria compared with wild type apidaecin-HbIb, such as the reported apidaecin 1C-20, which has the strongest anti-bacterial ability [23]. Additionally, the increase in N-terminal proline could increase its non-lytic ability and reduce toxicity to human cells [43]. Hence, the present study found that a novel secreting apidaecin Api-PR19 could retain major antimicrobial activity under stress challenge (low pH, high temperature, and protease treatment), potentially due to the amino acid composition of Api-PR19 with the addition of a proline to the N-terminus of apidaecin 1C-20. Basically, the present study further identified the antimicrobial activity of Api-PR19 and its potential roles in replacing AGPs in the food animal industry.

In a previous study, the AGPs were widely used in the food animal industry mainly due to their beneficial effects of increased growth performance, promoted immune function and inhibition of pathogenic bacteria [12, 44], as also indicated in the present study by considering the significantly decreased ratio of feed intake to weight gain, significantly increased intestinal sIgA content, and significantly decreased *Escherichia coli* and *Campylobacter jejuni*. In a previous study, the growth-promoting effect of antibiotics was mainly induced by a decreased risk of pathogenic infection and energy consumption of the underlying inflammatory response, as well as decreased energy loss due to microbial utilization [45]. Our results indicated that the use of antibiotics could actually provide these benefits but also revealed hidden dangers, including decreases in antibody levels against H9 avian influenza, harmful effects on intestinal morphology, and decreases in total bacteria. In previous studies, decreases in antibody levels against H9 avian influenza could increase the risk of infection with avian influenza [46], and the destroyed intestinal epithelia could cause further harm to nutrient absorption and induce intestinal inflammation [47, 48]. Specifically, according to previous studies, the first 2 disadvantages could be induced by the decrease in total bacteria, indicating a potentially decrease in probiotics in total bacteria [49], as suggested in a previous study. In comparison, apidaecin Api-PR19 could improve the feed conversion rate and improve immune function and defense against pathogenic bacteria like antibiotics. In contrast to the mechanism of antibiotics, the roles of apidaecin in promoting broiler growth were mainly due to their beneficial effects of improved intestinal absorption, including an improved duodenal and jejunal morphology and increased expression level of GLUT2, rBAT, y^+^LAT2, and CAT1 in the duodenum and jejunum, which respond to the amino acid and glucose transporter [27]. Moreover, increasing evidence suggests that ABPs protect hosts from bacteria via alternative mechanisms that are not related to their direct antimicrobial activity. It is well documented that ABPs are effector molecules of innate and adaptive immunity with the modulation of pro- and anti-inflammatory responses, chemotactic activity, and direct effects on adaptive immunity [50, 51], which could also be demonstrated by the significantly increased intestinal sIgA content in the present study. Moreover, the abundance of total bacteria was not influenced by ABP supplementation, which indicated that the increases in nutrient absorption were not induced by the decreased energy loss due to microbial utilization but might be influenced by an improved gut microbiota and subsequent improvement of the intestinal immune and developmental conditions [52].

The different roles of antibiotics and apidaecin in regulating the gut microbiota were further detected herein. In contract to apidaecin treatment, antibiotic treatment efficiently but indiscriminately ablated virtually all the gut bacteria, consistent with previous studies [45, 53]. In brief, a higher sterilization efficiency *in vitro* and a greater reduction of total bacteria and *Campylobacter jejuni* have confirmed this phenomenon. However, the indiscriminate effects of enramycin, including targeting of anaerobic commensal bacteria, leads to dysbiosis and suppression of colonization resistance, which could infer from the significant increase in the phylum of Proteobacteria and class of Deltaproteobacteria in the AGP group compared with the ABP group. In brief, Proteobacteria have been widely suggested as the key microbial signature of dysbiosis in the gut microbiota and a common factor in human diseases [54, 55]. Moreover, the expansion of Proteobacteria also serves as a microbial signature of epithelial dysfunction [56], which could also be demonstrated by the harmful effect of AGP and beneficial effect of ABP on intestinal morphology in the present study. Moreover, this dysbiosis consequently results in relapse of, and susceptibility to, other infections [57]. In comparison, in contrast to AGP supplementation, which improved growth performance but disrupted gut development, the gut microbiota, and health compared with the CON group, the RDA analysis indicated that ABP supplementation was simultaneously beneficial to growth performance and intestinal immune function, while it did not disturb the colonization of gut beneficial microbiota or, consequently, damage the intestinal morphology, supporting the advantage of ABP in replacing AGPs in broiler feeding [18]. In previous studies, the distribution of intestinal microbiota induced by antibiotics was mainly attributed to two differential main reasons, including the nonselective sterilization effects of AGPs and the antimicrobial resistance of some conditioned pathogens, which could separately result in reduced probiotics and increased unhealthy bacteria [45, 58]. However, compared with AGP, there is a narrower spectrum of antimicrobial agents but more efficient ability to kill conditioned pathogens, which could decrease unhealthy bacteria but not cause too much damage to probiotics. Moreover, low level-induced resistance to ABPs was also identified in a previous study [59, 60]. In particular, a major strength of ABPs is their ability to kill multidrug-resistant bacteria. Specifically, a recent study has shown that ABPs with characteristics of increased hydropathicity and fewer polar and positively charged amino acids are less prone to resistance in adaptive laboratory experiments [60]. Meanwhile, the increase in N-terminal proline in apidaecins could also increase its lethal ability on the Gram-negative bacteria, which indicated that the apidaecin Api-PR19 is more selective for Gram positives bacteria [43]. Additionally, the physicochemical features of apidaecin Api-PR19 was absolutely in accordance with these characteristics, contributing to the beneficial roles of apidaecin Api-PR19 in improving the intestinal microbiota, including the increased abundance of Gram positives bacteria in gut when using the apidaecin Api-PR19 in the present study.

The alteration of the gut microbiota could further result in beneficial or harmful effects on growth, immune function and intestinal function of the host [61–63]. Hence, in the present study, Spearman correlation analyses were performed to identify the beneficial or harmful roles of several key significantly changed bacteria induced by the antibiotics or apidaecin Api-PR19 in regulating growth performance and intestinal development and immune function. Together with the results obtained for the differential microbiota induced by AGP and ABP, we found that the increased bacteria in the ABP group were positively correlated with intestinal sIgA content and intestinal villus height, including the genera of *Eubacterium* and *Christensenella* and species of *uncultured*_*Eubacterium*_*sp*, *Clostridium_asparagiforme*, and *uncultured_Christensenella_sp*. Moreover, ABP supplementation could significantly decrease the abundance of *Prevotella_lascolaii*, *Helicobacter*_*cf*._*pullorum*, *Clostridium*_*colinum*, *Holdemania*_*filiformis*, *Anaeromassilibacillus*_*sp*._*Marseille*-*P4683*, *Prevotella*_*lascolaii*, and *Faecalibacterium*_*prausnitzii*, which were negatively correlated with growth performance, intestinal sIgA content and intestinal villus height, again confirming that ABP could be beneficial to growth, intestinal immune and development progress [64]. However, the increased bacteria in the AGP group, including *Prevotella*, *Tyzzerella*, and *Bacillaceae* and the species of *Bacillaceae*_*2*_*unclassified* and *Sporobacter*_*termitidis*, were almost negatively correlated with growth performance and intestinal morphology and sIgA content, which again indicated that AGP could increase these bacteria and further damage the intestinal condition and function [65]. Overall, compared with the harmful effects of antibiotics on microbial dysbiosis, apidaecin Api-PR19 could synergize with the above increased beneficial bacteria to combat conditioned pathogens, maintain intestinal health, and promote growth performance and immune functions in broilers.

At last, the roles of changed gut microbiota, which regulated by the dietary apidaecin Api-PR19 supplementation, in maintaining intestinal health and promoting growth performance of broilers have been suggested in the present study. However, due to the experimental limitation induced by that only 5 replicates were used for 16S rRNA gene sequencing in the present study, there could be other factors contributing to the observed differences in microbiota alteration, including the environmental effects and pen effects. Hence, further experiments with a larger population are suggested to perform, with aim to further exam the regulatory effect of apidaecin Api-PR19 on the gut microbiota. Meanwhile, the roles of a single changed bacteria in maintaining intestinal health and promoting growth performance could be also further verified in the future.

## Conclusion

The apidaecin Api-PR19 could combat pathogen infection and had a reduced impact on nontargeted bacteria in the broiler gut than antibiotic treatment. The beneficial bacteria and microbiota in broilers were not disturbed but improved by apidaecin Api-PR19, including the genera of *Eubacterium* and *Christensenella* and species of *uncultured*_*Eubacterium*_*sp*, *Clostridium*_*asparagiforme*, and *uncultured*_*Christensenella*_*sp*, which subsequently improved intestinal development, absorption, and immune function.

## Supplementary information

**Additional file 1: Figure S1.** The differential bacteria among CON, AGP, and ABP identified using the linear discriminant analysis and effect size (LEfSe) analysis. Note: CON indicates control group; ABP indicates Api-PR19 group; AGP indicates enramycin (antibiotic) group. The differential microbiota at each level (including phylum, class, family, genus and species) were analyzed and the first letter before the bacteria represents the level of the identified bacteria: p: phylum, c: class, f: family, g: genus, s: species. **Table S1.** Physical and chemical parameters of apidaecin-HbIb, apidaecin-Hb1C-20, and api-PR19. **Table S2.** Composition and nutrient level of the basal diet (air-dry basis). **Table S3.** Broiler immunization program used in the present study. **Table S4.** Primers used in the relative real-time quantitative PCR for gene expression in broilers. **Table S5.** Primers used in the absolute real-time quantitative PCR for caecal bacteria in broilers. **Table S6.** The effect of Api-PR19 on villus height, crypt depth and villus height/crypt depth ratio in the duodenum, jejunum, and ileum of broiler chickens.

## Data Availability

The sequence data were deposited and are available in the Sequence Read Archive (SRA) of NCBI under accession project number PRJNA578221.

## Abbreviations

LDA: Linear discriminant analysis
LEfSe: Linear discriminant analysis effect size
OTUs: Operational taxonomic units
PCoA: Principal coordinated analysis
qRT-PCR: Quantitative real-time PCR
sIgA: Secreted immunoglobulin A
