# Supplementary material for: A novel apidaecin Api-PR19 synergizes with the gut microbial community to maintain intestinal health and promote growth performance of broilers
Source: J Anim Sci Biotechnol. 2020 Jun 17;11:61. doi: 10.1186/s40104-020-00462-1 (PMC7298829; doi:10.1186/s40104-020-00462-1)
Supplement: Supplementary file 1 — Additional file 1: Figure S1. The differential bacteria among CON, AGP, and ABP identified using the linear discriminant analysis and effect size (LEfSe) analysis. Note: CON indicates control group; ABP indicates Api-PR19 group; AGP indicates enramycin (antibiotic) group. The differential microbiota at each level (including phylum, class, family, genus and species) were analyzed and the first letter before the bacteria represents the level of the identified bacteria: p: phylum, c: class, f: family, g: genus, s: species. Table S1. Physical and chemical parameters of apidaecin-HbIb, apidaecin-Hb1C-20, and api-PR19. Table S2. Composition and nutrient level of the basal diet (air-dry basis). Table S3. Broiler immunization program used in the present study. Table S4. Primers used in the relative real-time quantitative PCR for gene expression in broilers. Table S5. Primers used in the absolute real-time quantitative PCR for caecal bacteria in broilers. Table S6. The effect of Api-PR19 on villus height, crypt depth and villus height/crypt depth ratio in the duodenum, jejunum, and ileum of broiler chickens. [file 40104_2020_462_MOESM1_ESM.docx]

A novel apidaecin Api-PR19 synergizes with the gut microbial community to maintain intestinal health and promote growth performance of broilers

Shengru Wu^*，1^, Jian Wang^*^, Liqin Zhu^*^, Hao Ren^*^, Xiaojun Yang^*, 1^

* College of Animal Science and Technology, Northwest A&F University, Yangling, Shaanxi, China

Running Head: Apidaecin and gut microbiota

**^1^ Corresponding author:**

Shengru Wu, email: wushengru2013@163.com (S.R. Wu);

Xiaojun Yang, email: yangxj@nwsuaf.edu.cn (X. J. Yang); fax +86 29 87092164; Tel.: +86 13319284469.

**Email addresses of other authors:**

Shengru Wu: wushengru2013@163.com, Jian Wang: poultrywang@nwafu.edu.cn, Liqin Zhu: 954208918@qq.com, Hao Ren: 18729502782@163.com.

**Supplementary information**

**Figure S1 The differential bacteria among CON, AGP, and ABP identified using the linear discriminant analysis and effect size (LEfSe) analysis.** Note: CON indicates control group; ABP indicates Api-PR19 group; AGP indicates enramycin (antibiotic) group. The differential microbiota at each level (including phylum, class, family, genus and species) were analyzed and the first letter before the bacteria represents the level of the identified bacteria: p: phylum, c: class, f: family, g: genus, s: species.

**Table S1 Physical and chemical parameters of apidaecin-HbIb, apidaecin-Hb1C-20, and api-PR19.**

**Table S2 Composition and nutrient level of the basal diet (air-dry basis).**

**Table S3 Broiler immunization program used in the present study.**

**Table S4 Primers used in the relative real-time quantitative PCR for gene expression in broilers.**

**Table S5 Primers used in the absolute real-time quantitative PCR for caecal bacteria in broilers.**

**Table S6 The effect of Api-PR19 on villus height, crypt depth and villus height/crypt depth ratio in the duodenum, jejunum, and ileum of broiler chickens.**

**
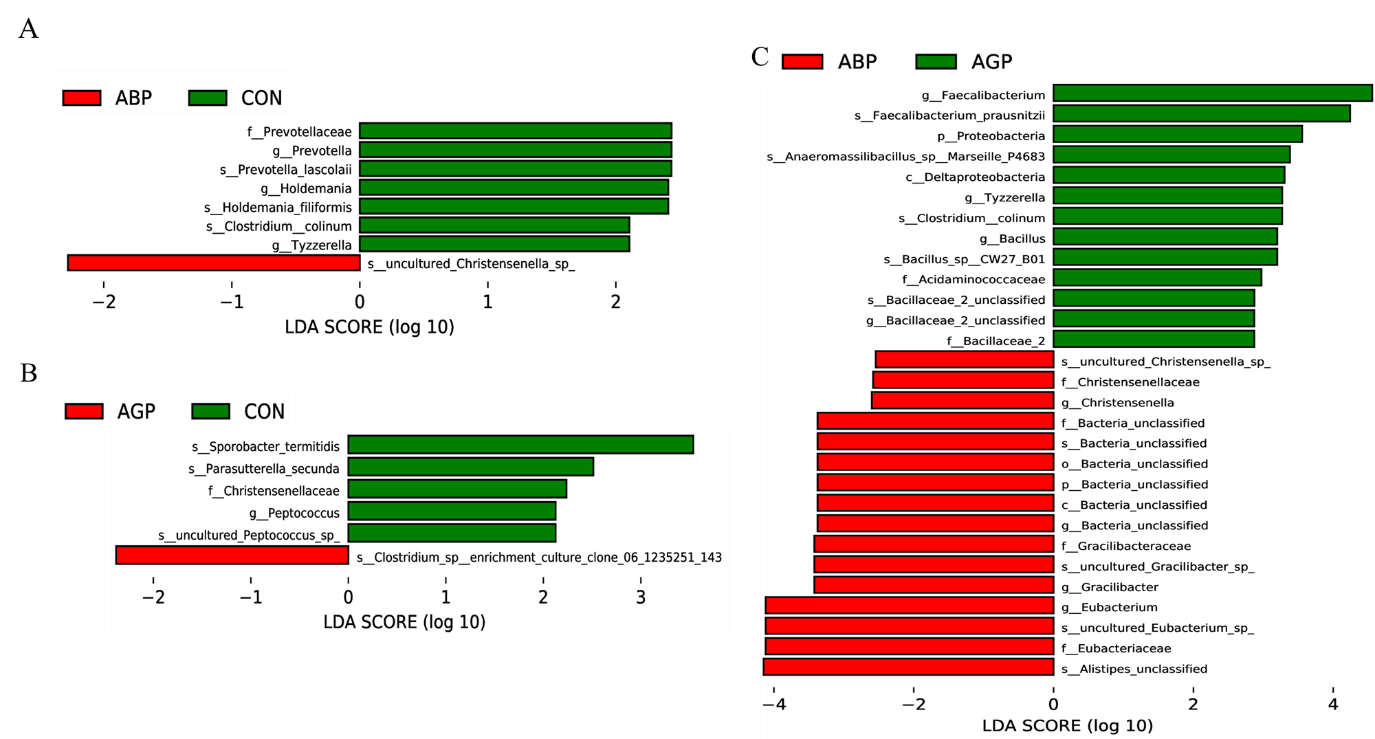
**

**Figure S1 The differential bacteria among CON, AGP, and ABP identified using the linear discriminant analysis (LDA) and effect size (LEfSe) analysis.**

**Table S1 Physical and chemical parameters of apidaecin-HbIb, apidaecin-Hb1C-20, and api-PR19.**

| Item | Apidaecin | | |
| --- | --- | --- | --- |
|  | Apidaecin-HbIb | Apidaecin-Hb1C-20 | Api-PR19 |
| Amino acid composition | GNNRPVYIPQPRPPHPRL | PRVRRPVYIPQPRPPHPRL | PRVRRPVYIPQPRPPHPRL |
| Isoelectric point | 11.71 | 12.18 | 12.18 |
| Charge number (+) | 4 | 5 | 5 |
| Total hydrophilic index | -1.444 | -1.300 | -1.316 |
| Coefficient of fat | 59.44 | 75.56 | 71.58 |
| Beaumont index, kcal/mol | 2.97 | 3.72 | 3.52 |
| Antibacterial activity score | 1.174 | 0.692 | 0.802 |

**Table S2 Composition and nutrient level of the basal diet (air-dry basis).**

| Ingredients*,* % | 1 to 3 weeks | 4 to 6 weeks |  | Nutrient levels*^2^* | 1 to 3 weeks | | 4 to 6 weeks | |
| --- | --- | --- | --- | --- | --- | --- | --- | --- |
| Corn | 53.61 | 59.80 |  | ME, MJ/kg | 12.24 | | 12.87 | |
| Soybean meal | 22.82 | 15.52 |  | CP, % | 21.20 | | 19.30 | |
| DDGS | 5.00 | 6.00 |  | EE, % | 2.61 | | 6.39 | |
| Flour | 2.00 | 2.50 |  | Ca, % | 0.85 | | 0.85 | |
| Corn gluten meal | 4.00 | 4.00 |  | Total P, % | 0.68 | | 0.62 | |
| Lard | 2.30 | 3.20 |  | Available P, % | 0.43 | | 0.35 | |
| Corn germ meal | 3.00 | — |  | Lys, % | 1.29 | | 1.27 | |
| Peanut meal | 2.50 | 4.00 |  | Met, % | 0.60 | | 0.65 | |
| CaHCO_3_ | 1.76 | 1.30 |  |  |  | |  | |
| Ca power | 0.90 | 1.21 |  |  |  | |  | |
| L-Lysine sulphate | 0.75 | 1.01 |  |  |  | |  | |
| Salt | 0.25 | 0.25 |  |  |  | |  | |
| DL-Met | 0.28 | 0.36 |  |  |  | |  | |
| Thr | 0.15 | 0.26 |  |  |  | |  | |
| Limestone | 0.20 | 0.20 |  |  |  | |  | |
| Choline chloride | 0.08 | 0.10 |  |  | |  | |  |
| Premix | 0.19 | 0.19 |  |  | |  | |  |
| Tryptophan | 0.01 | — |  |  | |  | |  |
| Sodium bicarbonate | 0.20 | 0.10 |  |  | |  | |  |
| Total | 100.00 | 100.00 |  |  | |  | |  |

**Table S3 Broiler immunization program used in the present study.**

| Age | Type of vaccine | Method |
| --- | --- | --- |
| 1 d | Chicken marek's disease vaccine | Cervical subcutaneous injection |
| 3 d, extra vaccine for immunization | Avian influenza (H5N1 Re-5+H9N2 Re-2 bivalent inactivated vaccine) | Intramuscular injection |
| 7 d | New city disease + infectious bronchitis | Eye droppings, nasal inhalation |
| 14 d | Chicken marek's disease vaccine | Drinking water |
| 21 d | New city disease + avian influenza | Intramuscular injection |

**Table S4 Primers used in the relative real-time quantitative PCR for gene expression in broilers.**

| Gene | Sequence of Primers from 5’ to 3’ |
| --- | --- |
| *β-actin* | F: ACTCCTACGGGAGGCAGCAGT  R: ATTACCGCGGCTGCTGGC |
| *SGLT1* | F: AGCATTTCAGCATGGTGTGTCTTC  R: GATGCTCCTATCTCAGGGCAGTTC |
| *GLUT2* | F: CACACTATGGGCGCATGCT  R: ATTGTCCCTGGAGGTGTTGGTG |
| *rBAT* | F: CCCGCCGTTCAACAAGAG  R: AATTAAATCCATCGACTCCTTTGC |
| *y^+^LAT2* | F: GCCCTGTCAGTAAATCAGACAAGA  R: TTCAGTTGCATTGTGTTTTGGTT |
| *CAT1* | F: CAAGAGGAAAACTCCAGTAATTGCA  R: AAGTCGAAGAGGAAGGCCATAA |

**Table S5 Primers used in the absolute real-time quantitative PCR for caecal bacteria in broilers.**

| Item | Size, bp | Primers Sequence from 5’ to 3’ |
| --- | --- | --- |
| Total bcateria | 172 | F: ACTCCTACGGGAGGCAGCAGT  R: ATTACCGCGGCTGCTGGC |
| *Escherichia coil* | 96 | F: CATGCCGCGTGTATGAAGAA  R: CGGGTAACGTCAATGAGCAAA |
| *Bifidobacterium bifidum* | 290 | F: GGAAGAACACCGATGGCGAAG  R: ATGTCAAGCCCAGGTAAGG |
| *Salmonella gallinarum* | 236 | F: GGGTGAGTAATGTCTGGGAAAC  R: CGTAGGAGTCTGGACCGTGT |
| *Campylobacter jejuni* | 251 | F: AGACACGGTCCAGACTCCTA  R: GACTTGATAATCCGCCTACG |

**Table S6 The effect of Api-PR19 on villus height, crypt depth and villus height/crypt depth ratio in the duodenum, jejunum, and ileum of broiler chickens.**

| Intestine | Age | | Item | CON | AGP | Api-PR19, mg/kg | | | SEM | *P-*value |
| --- | --- | --- | --- | --- | --- | --- | --- | --- | --- | --- |
|  |  |  |  |  |  | 100 | 200 | 300 |  |  |
| Duodenum | 21 d | villus height, μm | | 1715.41 | 1683.06 | 1864.59 | 1909.45 | 2010.36 | 59.37 | 0.398 |
|  |  | crypt depth, μm | | 146.16 | 142.41 | 127.47 | 112.55 | 123.38 | 6.11 | 0.414 |
|  |  | villus height/crypt depth | | 11.81 | 12.44 | 15.18 | 18.04 | 17.68 | 1.01 | 0.160 |
|  | 42 d | villus height, μm | | 1560.16^b^ | 1549.5^b^ | 1868.29^ab^ | 1929.30^a^ | 1709.98^ab^ | 47.08 | 0.013 |
|  |  | crypt depth, μm | | 240.24 | 142.35 | 140.10 | 148.99 | 138.70 | 13.80 | 0.072 |
|  |  | villus height/crypt depth | | 7.87^b^ | 10.87^ab^ | 13.37^ab^ | 13.97^a^ | 13.25^ab^ | 0.76 | 0.050 |
| Jejunum | 21 d | villus height, μm | | 1227.66^ab^ | 944.83^b^ | 1297.13^ab^ | 1597.23^a^ | 1636.92^a^ | 68.37 | 0.002 |
|  |  | crypt depth, μm | | 143.67 | 123.07 | 127.21 | 130.31 | 134.83 | 4.00 | 0.571 |
|  |  | villus height/crypt depth | | 8.59^bc^ | 7.70^c^ | 10.23^ab^ | 12.25^a^ | 12.14^a^ | 0.47 | 0.002 |
|  | 42 d | villus height, μm | | 1627.17 | 1673.86 | 1570.89 | 1669.34 | 1776.98 | 38.54 | 0.577 |
|  |  | crypt depth, μm | | 119.45 | 120.87 | 129.16 | 122.08 | 103.75 | 4.31 | 0.467 |
|  |  | villus height/crypt depth | | 13.90 | 14.13 | 12.60 | 14.01 | 17.74 | 0.67 | 0.148 |
| Ileum | 21 d | villus height, μm | | 1274.82 | 1104.87 | 1098.65 | 1181.91 | 1394.98 | 53.00 | 0.366 |
|  |  | crypt depth, μm | | 136.60 | 144.41 | 144.04 | 136.52 | 132.68 | 3.86 | 0.185 |
|  |  | villus height/crypt depth | | 9.36 | 7.99 | 7.69 | 8.89 | 10.53 | 0.46 | 0.864 |
|  | 42 d | villus height, μm | | 691.62 | 789.05 | 772.24 | 874.12 | 749.47 | 35.62 | 0.630 |
|  |  | crypt depth, μm | | 125.07 | 120.92 | 116.66 | 135.24 | 114.39 | 4.95 | 0.854 |
|  |  | villus height/crypt depth | | 5.96 | 6.57 | 6.67 | 6.63 | 6.75 | 0.36 | 0.968 |
